# Bradykinin inhibits oxidative stress-induced senescence of endothelial progenitor cells through the B2R/AKT/RB and B2R/EGFR/RB signal pathways

**DOI:** 10.18632/oncotarget.5071

**Published:** 2015-08-24

**Authors:** Cong Fu, Bing Li, Yuning Sun, Genshan Ma, Yuyu Yao

**Affiliations:** ^1^ Department of Cardiology, Zhongda Hospital, Medical School of Southeast University, Nanjing, Jiangsu 210009, China

**Keywords:** endothelial progenitor cells, B2 receptor, bradykinin, senescence

## Abstract

Circulating endothelial progenitor cells (EPCs) have multiple protective effects that facilitate repair of damage to tissues and organs. However, while various stressors are known to impair EPC function, the mechanisms of oxidative stress-induced EPC senescence remains unknown. We demonstrated that B2 receptor (B2R) expression on circulating CD34^+^ cells was significantly reduced in patients with diabetes mellitus (DM) as compared to healthy controls. Furthermore, CD34^+^ cell B2R expression in patients with DM was inversely correlated with plasma myeloperoxidase concentrations. Bradykinin (BK) treatment decreased human EPC (hEPC) senescence and intracellular oxygen radical production, resulting in reduced retinoblastoma 1 (RB) RNA expression in H_2_O_2_-induced senescent hEPCs and a reversal of the B2R downregulation that is normally observed in senescent cells. Furthermore, BK treatment of H_2_O_2_-exposed cells leads to elevated phosphorylation of RB, AKT, and cyclin D1 compared with H_2_O_2_-treatment alone. Antagonists of B2R, PI3K, and EGFR signaling pathways and B2R siRNA blocked BK protective effects. In summary, this study demonstrates that BK significantly inhibits oxidative stress-induced hEPC senescence though B2R-mediated activation of PI3K and EGFR signaling pathways.

## INTRODUCTION

Endothelial progenitor cells (EPCs) are adult stem cells that exist within the vascular circulation and have numerous protective effects due to their ability to differentiate into endothelial cells, repair vascular intima injury, and promote angiogenesis post myocardial infarction [1]. However, stress-induced premature (SIP) senescence of EPCs caused by oxidative stress related to coronary artery disease and diabetes can impair their function, leading to harmful outcomes. Stress-induced premature senescence is characterized by DNA damage, resulting in dysfunction, inhibition of mitosis, senescence, and even apoptosis. In addition, research has shown that decreases in the quantity and quality of EPCs can lead to enhanced progression of diabetes [2].

Numerous studies on the mechanisms of cellular senescence utilizing tumor cells and immortalized cell lines have revealed that retinoblastoma 1 (RB) plays a key role in cell senescence by suppressing the expression of genes involved in DNA replication [3]. However, while oxidative stress can induce cell senesence via the RB signaling pathway, the mechanism of EPC senescence induced by oxidative stress remains unknown [4, 5].

The tissue kallikrein-kinin system (KKS) has essential regulatory functions that can depress blood pressure and counter oxidative stress that have been validated in previous research [6]. The peptide bradykinin (BK) is a potent vasodilator and inflammatory mediator that can protect endothelial and myocardial cells against inflammation and ischemia injury via the B2 receptor (B2R). Previous research from our laboratory has shown that EPCs promote angiogenesis following myocardial infarction through the B2R signaling pathway [7]. Recently, other studies have demonstrated that BK suppresses cell DNA damage in a manner that has the potential for eliciting anti-senescence effects [8–11]. However, whether or not BK protects EPCs against oxidative stress-induced senescence via the B2R remains unknown.

This study was designed to confirm the anti-senescence effects of BK signaling via the B2R pathway in EPCs and to investigate the detailed regulatory mechanism controlling this activity, which has important implications for the treatment of coronary artery disease and diabetes.

## RESULTS

### B2R expression on circulating CD34^+^ cells from patients with diabetes mellitus (DM) and healthy controls

**Figure 1:**
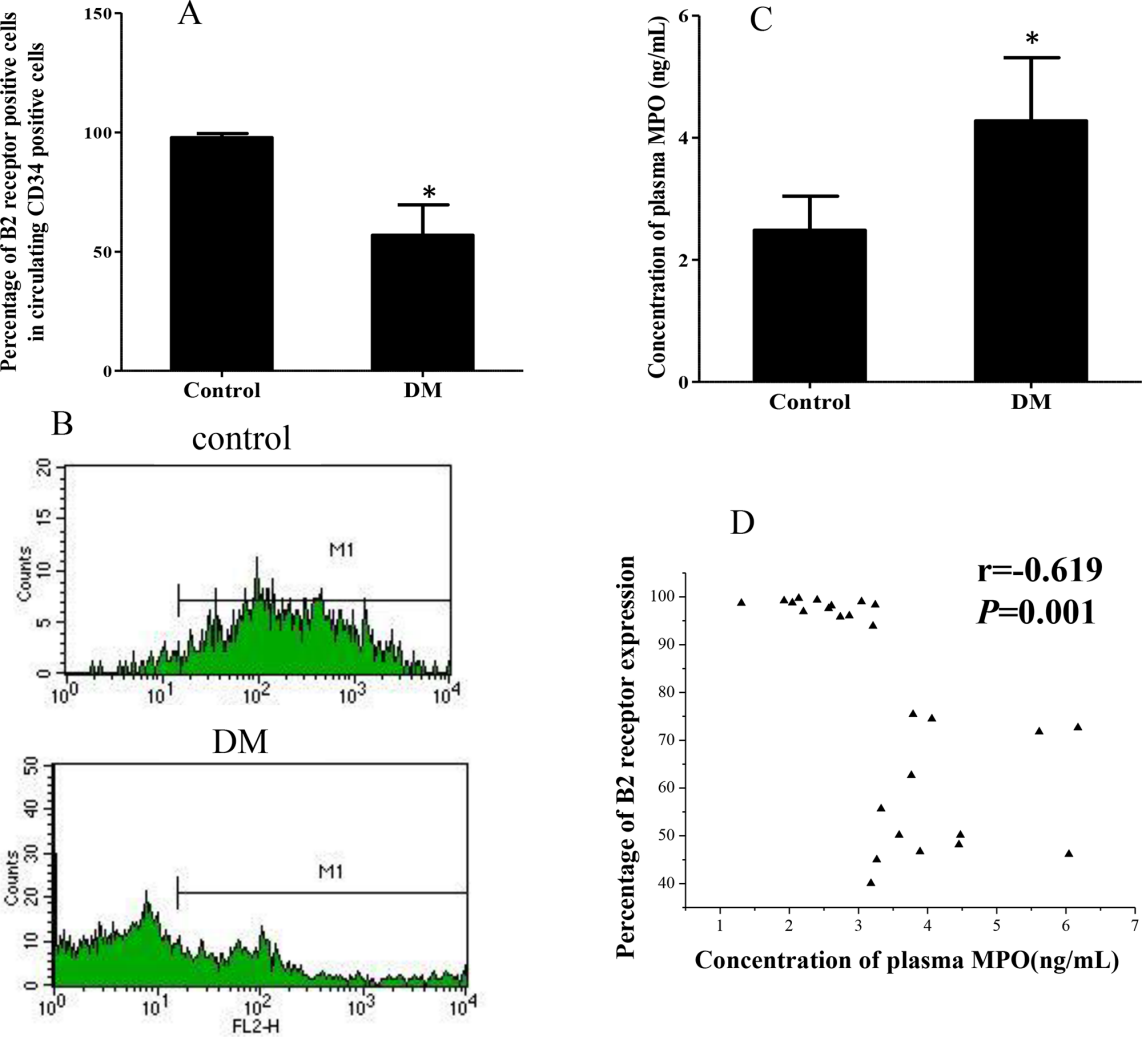
Expression of B2R on circulating CD34 positive cells of DM patients and healthy controls **A.** Graph showing that the percentage of circulating CD34 positive cells also immunopositive for B2R in DM patients (*n* = 13) was significantly lower than in healthy controls (*n* = 13). **B.** Representative flow cytometry analysis of B2R positive cells within the population of circulating CD34^+^ cells of both DM patients and healthy controls. M1 stand for B2R positive cells. **C.** Plasma MPO concentration of DM patients was significantly higher than healthy controls. **D.** Pearson correlation analyses showing the correlation of plasma MPO concentrations with B2R expression of CD34^+^ cells (*r* = − 0.619, *p* = 0.001) *p < 0.001; B2R: Bradykinin receptor 2. DM: Diabetes Mellitus. MPO: Plasma Myeloperoxidase.

No differences in age or gender were found between the DM and healthy control study groups. However, the expression of B2R on circulating CD34^+^ cells from DM patients was significantly reduced compared to healthy controls (Figures 1A and 1B; 56.8 ± 12.8% vs 97.8 ± 1.7%; *p* < 0.001). Furthermore, DM patient plasma myeloperoxidase (MPO) concentrations were significantly higher than for controls (Figure 1C; 4.3 ± 1.0 ng/mL vs 2.5 ± 0.6 ng/mL, *p* < 0.001). Pearson correlation analyses showed that plasma MPO concentration was inversely correlated with the B2R expression level on CD34^+^ cells (Figure 1D; *r* = −0.619; *p* = 0.001).

### Characterization of cultured hEPCs

**Figure 2:**
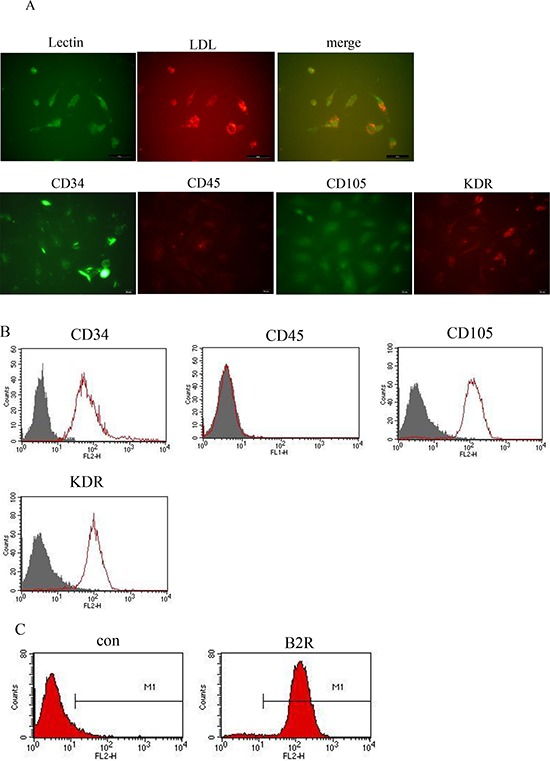
Phenotypic characterization of cultured hEPCs including analysis of B2R expression **A.** Photomicrographs showing that the adherent cells intensively took up acLDL-Dil and bound an endothelial-specific lectin, as assessed using fluorescence microscopy. (Original magnification: 400×). **B.** Representative flow cytometry analyses of hEPCs for expression of cell surface markers. The hEPCs of passage 3 were positive for CD34, KDR, and CD105, but were negative for CD45. **C.** Representative flow cytometry analysis of hEPCs for the expression of B2R. hEPCs: Human Endothelial Progenitor Cells. acLDL-Dil; acetylated low-density lipoprotein; KDR: vascular endothelial growth factor receptor.

Human umbilical cord blood-derived mononuclear cells (MNCs) were separated by density-gradient centrifugation. Double staining for FITC-lectin and acLDL-Dil showed that human EPCs (hEPCs) were able to uptake acLDL-Dil, which binds to an endothelial cell-specific lectin. Immunofluorescence showed that these hEPCs expressed CD34, kinase domain receptor (KDR), and CD105, but not CD45. hEPCs were immunopositive for CD34, KDR, CD105, and B2R, but not CD45 by flow cytometry (Figure 2).

### BK inhibits oxidative stress-induced senescence

**Figure 3:**
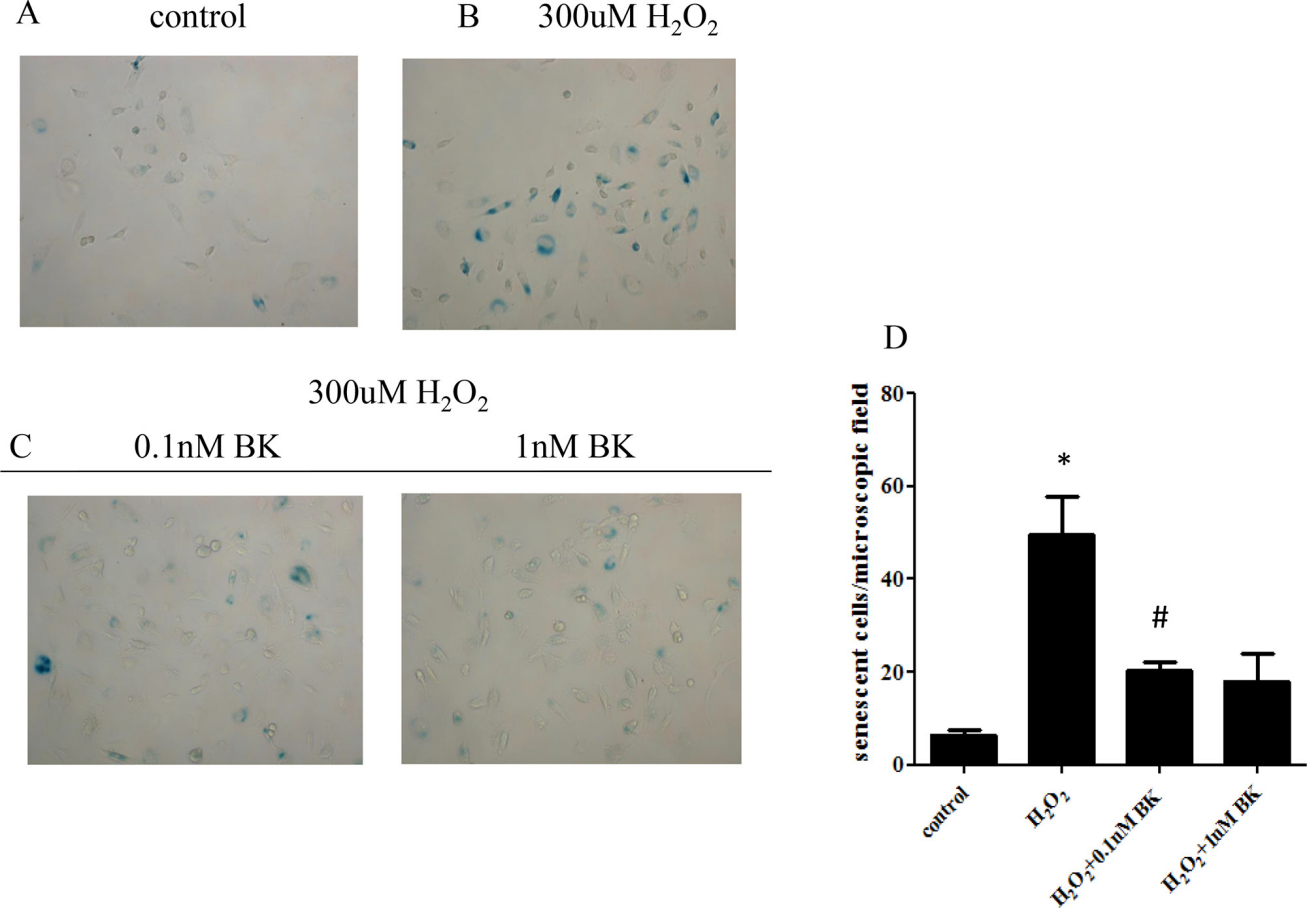
BK inhibits oxidative stress induced senescence of hEPCs **A.** SA-Gal staining showing the degree of senescence in untreated controls. **B.** SA-Gal staining showing senescent hEPCs following H_2_O_2_ induced oxidative stress. **C.** Effect of 0.1 nM and 1nM BK on SA-Gal staining of senescent hEPCs. **D.** Histograph showing the number of senescent cells per microscopic field. (Original magnification 200 ×; *n* = 5 for each group) demonstrating that both concentrations of BK significantly inhibit oxidative stress induced senescence in hEPCs. *p < 0.05 vs control and both concentrations of BK, ^#^*p* > 0.05 vs 1.0 nM BK) BK: Bradykinin; hEPCs: Human Endothelial Progenitor Cells; SA-Gal: β-galactosidase.

β-galactosidase (SA-Gal) staining revealed that 300 μM H_2_O_2_ significantly induced hEPC senescence (mean ± SEM, 49.6 ± 8.2 cells/field vs 6.4 ± 1.1 cells/field, *p* < 0.05). Furthermore both 0.1 nM and 1.0 nM BK dramatically inhibited H_2_O_2_-induced hEPC senescence compared to cells treated with H_2_O_2_ alone (20.4 ± 1.7 cells/field vs 6.4 ± 1.1 cells/field for 0.1 nM BK; 18.0 ± 6.0 cells/field vs 6.4 ± 1.1 cells/field for 1.0 nM BK; *p* < 0.05). No statistical difference in oxidative stress-induced senescence was found between 0.1 and 1.0 nM BK treatment groups (*p* > 0.05, Figure 3).

### BK suppresses H_2_O_2_-induced intracellular oxygen radical production

**Figure 4:**
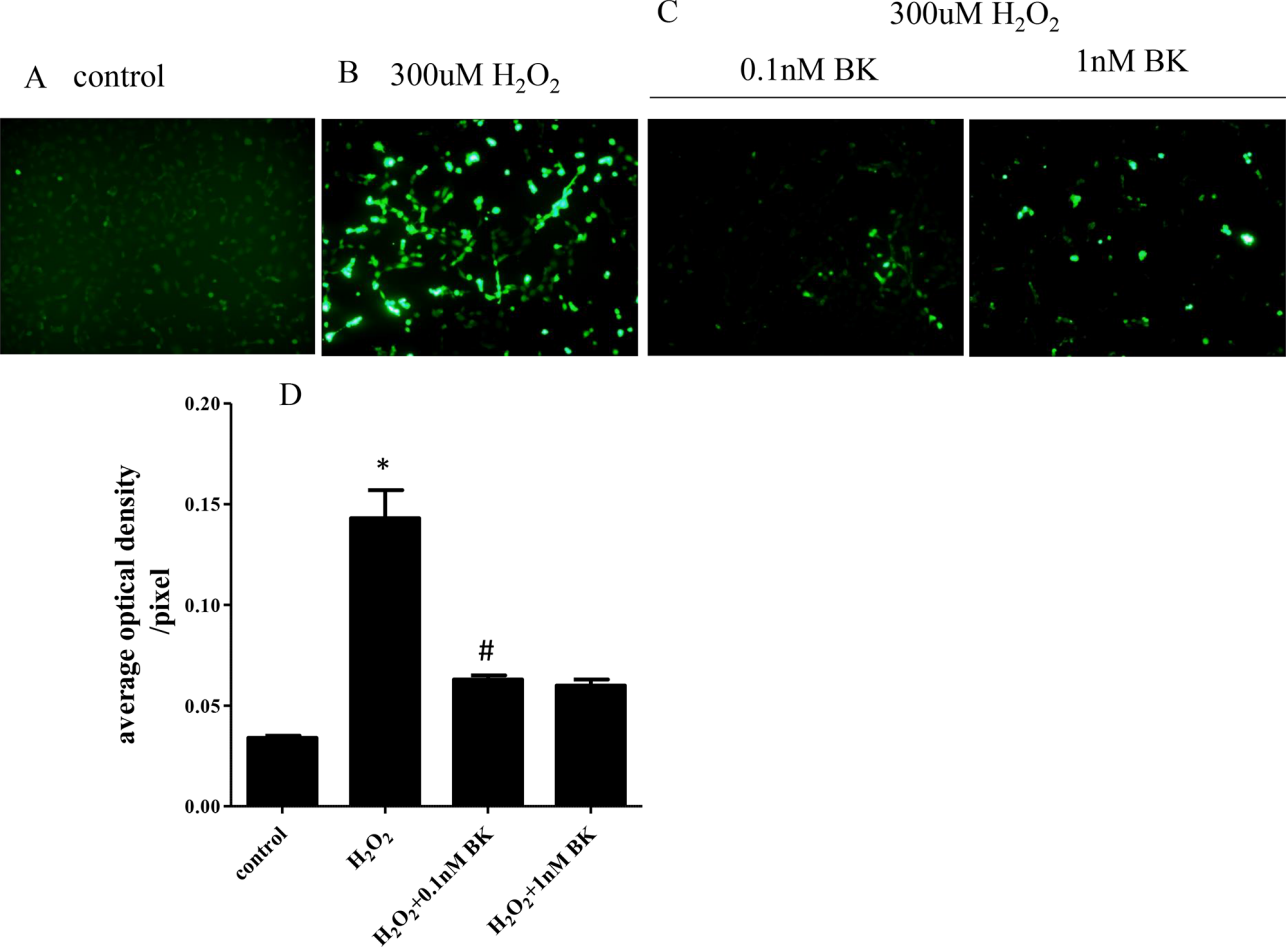
BK suppresses H_2_O_2_ induced production of intra-cellular free radicals **A.** Photomicrographs of untreated hEPCs labeled with DCFH-DA probe. **B.** hEPCs treated with 300 μM H_2_O_2_ and labeled with DCFH-DA probe demonstrating that H_2_O_2_ increases the production of intra-cellular oxygen radicals. **C.** hEPCs treated with 300 μM H_2_O_2_ and BK labeled with DCFH-DA probe illustrating 0.1nM and 1nM BK induced inhibition of intra-cellular oxygen radicals. Intra-cellular oxygen radicals positive cells are shown as green by fluorescence microscopy at an absorption wave length of 488 nm. **D.** Histogram showing the average optical density per pixel demonstrating that both concentrations of BK significantly inhibit generation of oxygen radicals in hEPCs (*n* = 5 for each group). *p < 0.05 vs control and 2 concentrations of BK, ^#^*p* > 0.05 vs 1.0 nM BK). BK: bradykinin; hEPCs: Human Endothelial Progenitor Cells; DCFH-DA: dichlorofluorescein diacetate.

Examination of intracellular oxygen radicals visualized using dichlorofluorescein diacetate (DCFH-DA) probes incubated with H_2_O_2_-induced senescent hEPCs showed that the senescent cells had significantly higher levels than normal controls (mean fluorescence intensities: 0.143 ± 0.014/pixel vs 0.034 ± 0.001/pixel, *p* < 0.05). Also, we found that treatment with BK at 0.1 nM (mean fluorescence intensities: 0.063 ± 0.002/pixel vs 0.143 ± 0.014/pixel, *p* < 0.05) and 1.0 nM (mean fluorescence intensities: 0.060 ± 0.003/pixel vs 0.143 ± 0.014/pixel, *p* < 0.05) suppressed the generation of intra-cellular oxygen radicals compared to hEPCs treated with H_2_O_2_ alone. No statistical differences were found between cells treated with the 2 concentrations of BK (*p* > 0.05, Figure 4).

### RB expression in H_2_O_2_-induced hEPC senescence was significantly decreased following BK treatment

**Figure 5:**
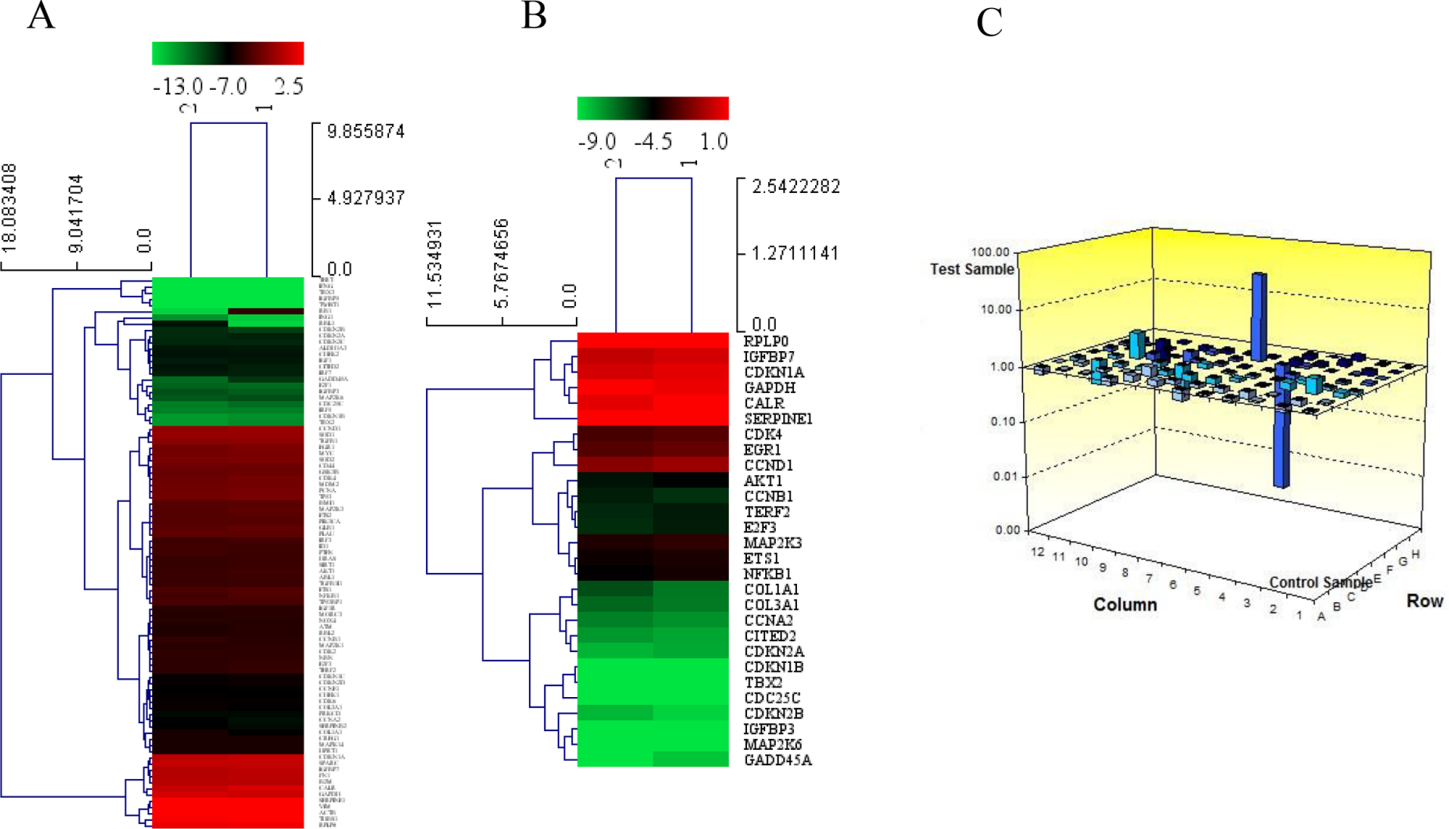
PCR array showing gene expression in cells treated with 300 μM H_2_O_2_ both with and without pretreatment with BK **A.** and **B.** Dendrogram illustrating all the genes expressed in hEPCs treated with 300 μM H_2_O_2_ both with and without pretreatment with BK show more than 1.2-fold difference between groups. **C.** Histogram showing the fold changes in gene and RB expression between the 2 groups. BK: Bradykinin; DM: Diabetes Mellitus.

As shown in Figure 5, a PCR array showed that treatment with 0.1 nM BK changed the expression of 33 senescence-associated genes more than 1.2-fold and 6 genes more than 2-fold (Supplemental Table 1). Notably, RB gene expression was down-regulated 176.15-fold after BK treatment.

### Signaling pathway inhibitors and B2R siRNA decrease the protective effects of BK

**Figure 6:**
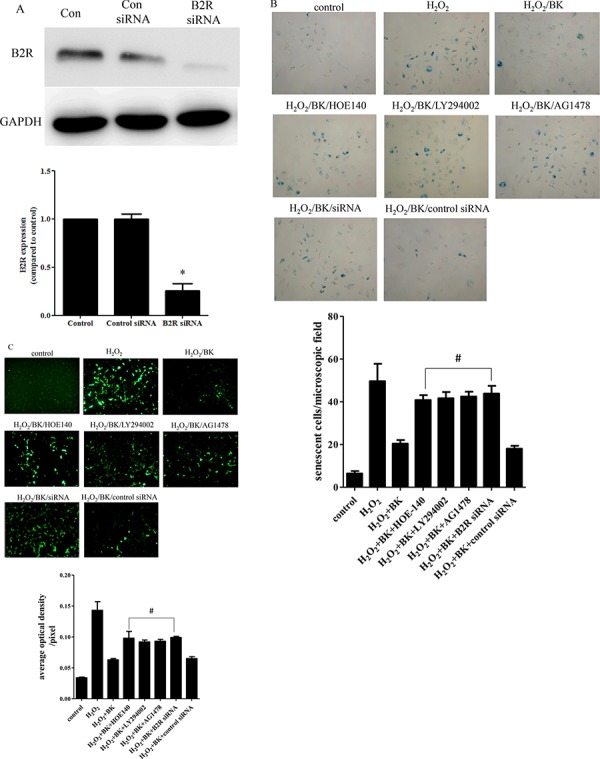
Signal pathway inhibitor and siRNA impair the protective effect of BK **A.** Western blot showing that B2R siRNA reduce the expression of B2R compared to control and control siRNA. Representative blots are shown in the upper panel and densitometry quantitation of protein expression levels are shown as fold changes in the lower panel (*n* = 5 for each group; *p < 0.05 vs control, control siRNA). **B.** Photomicrographs showing the effect of B2R antagonist HOE-140 (150 nM), PI3K antagonist LY-294002 (10 μM), EGFR antagonist AG1478 (10 μM), and B2R siRNA on the number of senescent hEPCs following treatment with 300 μM H_2_O_2_ and 0.1 nM BK (Original magnification 200×). Graph quantifying the effect of HOE-140, LY294002, AG1478, and B2R siRNA on the average senescent cells per field. **C.** Fluorescence photomicrographs showing that HOE-140 (150 nM), LY294002 (10 μM), AG1478 (10 μM) and B2R siRNA inhibit the effect of BK that suppress the generation of intra-cellular oxygen radicals. Graph quantifying the effect of HOE-140, LY294002, AG1478, and B2R siRNA on the average optical density per pixel. (Original magnification 200×) (*n* = 5 for each group; ^#^*p* < 0.05 vs H_2_O_2_ plus BK). BK: Bradykinin; hEPCs: Human Endothelial Progenitor Cells; B2R: Bradykinin 2 receptor.

Efficient B2R silencing was confirmed by a reduction in the B2R protein level as shown by Western blot (Figure 6A). HOE-140, LY-294002, AG1478, and B2R siRNA significantly antagonized the anti-senescence effects of BK in hEPCs compared with no inhibitor (40.8 ± 2.3 cells/field, 41.6 ± 3.0 cells/field, 42.4 ± 2.3 cells /field, and 43.8 ± 3.6 cells/field vs 20.4 ± 1.7 cells/field, respectively; *p* < 0.05; Figure 6B). Furthermore, incubating hEPCs treated with H_2_O_2_ and 0.1 nM BK with HOE-140, LY-294002, AG1478, and B2R siRNA significantly increased the generation of intracellular oxygen radicals in hEPCs than those treated with only H_2_O_2_ and 0.1 nM BK (mean fluorescence intensities: 0.098 ± 0.011/pixel, 0.092 ± 0.003/pixel, 0.093 ± 0.003/pixel, and 0.099 ± 0.002/pixel vs 0.063 ± 0.002/pixel, respectively; *p* < 0.05; Figure 6C)

### Expression of RB and B2R is regulated by BK in hEPCs subjected to oxidative stress

**Figure 7:**
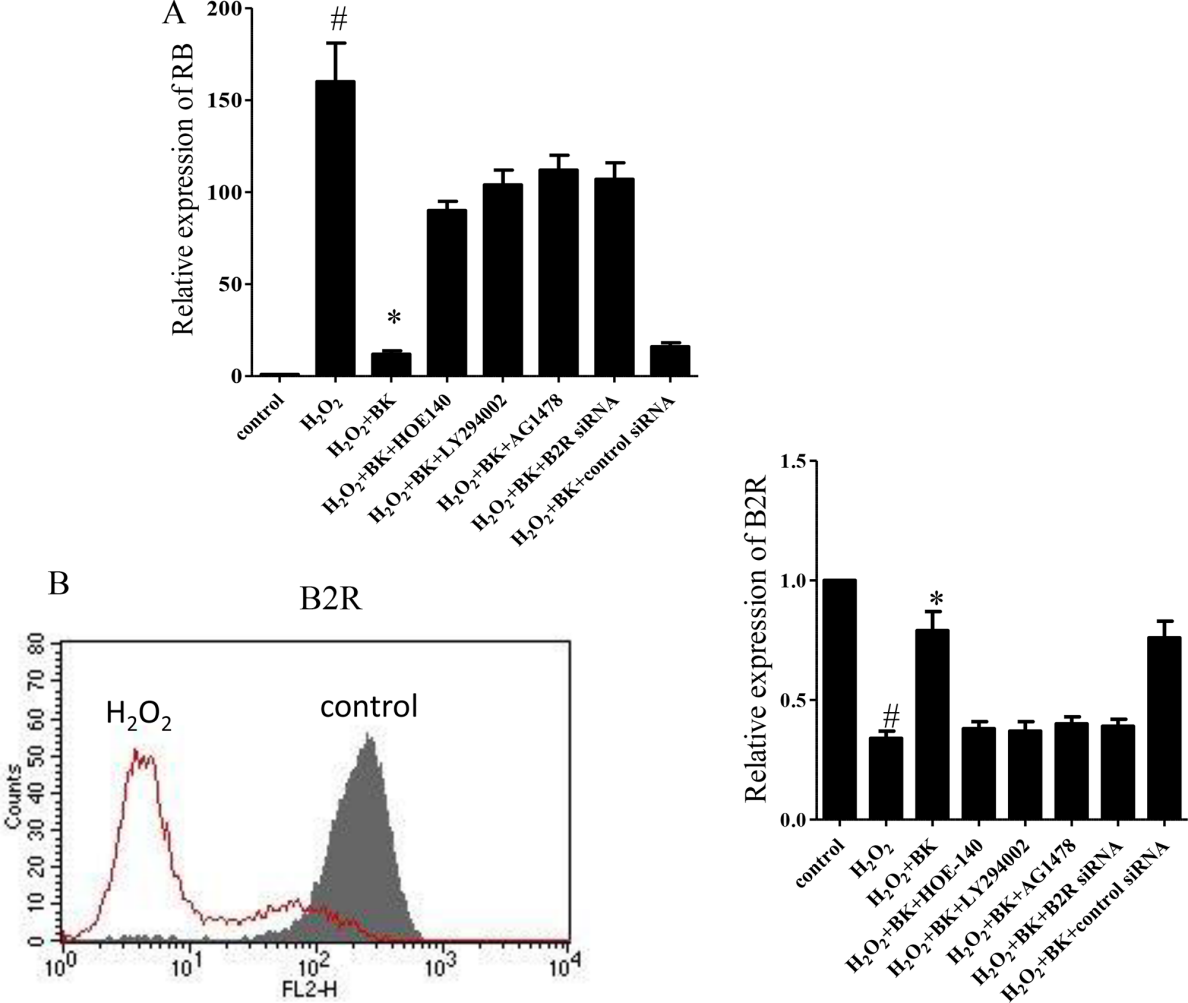
The effect of the B2R antagonist HOE-140 (150 nM), PI3K antagonist LY-294002 (10 μM), and EGFR antagonist AG1478 (10 μM), and B2R siRNA on the relative expression of RB and B2R in hEPCs treated with 300 μM H2O2 and BK **A.** Relative expression of RB in hEPCs confirmed by PCR. **B.** Representative flow cytometry analyses of hEPCs for expression of B2R with or without H_2_O_2_ treatment (left) and relative expression of B2R in hEPCs (right). (*n* = 5 for each group, *p < 0.05 vs control, H_2_O_2_ plus BK plus HOE-140, H_2_O_2_ plus BK plus LY294002, H_2_O_2_ plus BK plus AG1478 and H_2_O_2_ plus BK plus B2R siRNA. ^#^*p* < 0.05 vs controls). BK Bradykinin; hEPCs Human Endothelial Progenitor Cells; B2R Bradykinin 2 receptor; RB: Retinoblastoma 1.

For further validation of the results of the PCR array, qPCR was performed to measure RB expression. We found that RB expression was significantly reduced in hEPCs treated with H_2_O_2_ plus BK compared with those treated with H_2_O_2_ alone. However, when HOE-140, LY-294002, AG1478, and B2R siRNA were added to the culture media to block the signaling pathway, RB expression was up-regulated compared to those cells treated with H_2_O_2_ and BK alone. These data indicate that the effects of BK were impaired (Figure 7A). Additionally, the B2R expression by hEPCs treated with H_2_O_2_ was significantly lower than expression by normal controls, with BK treatment at least partially reversing this affect. Furthermore, treatment with HOE-140, LY-294002, AG1487, and B2R siRNA inhibited the effect of BK on H_2_O_2_-treated hEPCs (Figure 7B).

### BK prevents H_2_O_2_-induced hEPC senescence via the B2R/AKT/cyclin D1/RB and B2R/EGFR/cyclin D1/RB signaling pathways

**Figure 8:**
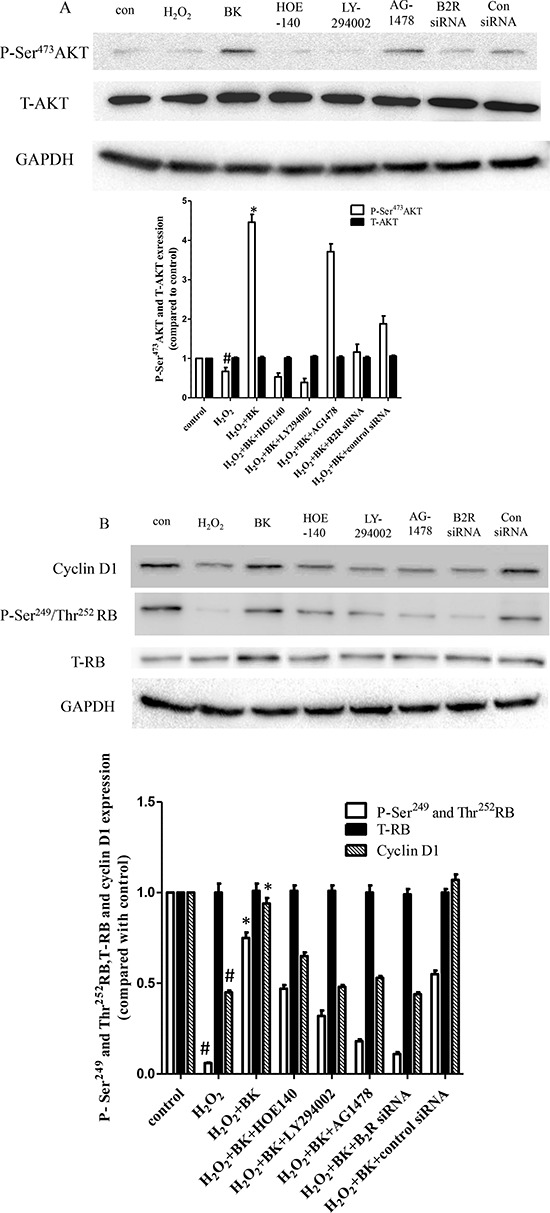
Western blots normalized to GAPDH showing that BK activates the phosphorylation of AKT and RB and increases cyclin D1 levels and the effect of B2R antagonist HOE-140 (150 nM), PI3K antagonist LY-294002 (10 μM), EGFR antagonist AG1478 (10 μM), and B2R siRNA **A.** BK activates the phosphorylation of AKT while the B2R, PI3K antagonists and siRNA diminish the effect of BK but EGFR antagonist have no effect on phosphorylation of AKT while total AKT levels remain constant. Representative blots are shown in the upper panel and densitometry quantitation of protein expression levels are shown as fold changes in the lower panel. **B.** BK activates the phosphorylation of RB and total cyclin D1 while the antagonists and siRNA inhibit the effect of BK while leaving total RB levels constant. Representative blots are shown in the upper panel and densitometry quantitation of protein expression levels are shown as fold changes in the lower panel. (*n* = 5 for each group, *p < 0.05 vs control, H_2_O_2_ plus BK plus HOE-140, H_2_O_2_ plus BK plus LY294002, H_2_O_2_ plus BK plus AG1478 and H_2_O_2_ plus BK plus B2R siRNA. #*p* < 0.05 vs control).

To determine the molecular signaling pathways that regulate hEPC senescence, the expression of AKT, P-Ser^473^AKT, RB, P-Ser^249^, Thr^252^RB, and cyclin D1 protein was detected by Western blot analysis. As shown in Figure 8A, P-Ser^473^AKT expression was down-regulated following treatment with 300 μM H_2_O_2_ compared to control cells. Furthermore, BK increased P-Ser^473^AKT expression in H_2_O_2_-treated hEPCs, while B2R blockade and siRNA knockdown, along with PI3K antagonist treatment, reduced P-Ser^473^AKT expression in H_2_O_2_-treated hEPCs compared to treatment with BK and H_2_O_2_ alone. However, the addition of AG1478 had no impact on P-Ser^473^AKT expression compared with BK treatment alone. Furthermore, there were no differences found in total AKT expression between groups, indicating that these results were due to changes in protein phosphorylation and not expression. Cyclin D1 expression was elevated in controls but reduced dramatically following H_2_O_2_-induced senescence. Also, while BK treatment up-regulated cyclin D1 expression, treatment with either a B2R antagonist or siRNA along with PI3K antagonist treatment reduced cyclin D1 and P-Ser^473^AKT expression compared with BK and H_2_O_2_ treatment alone. Treatment with AG1478 also reduced cyclin D1 expression. Additionally, we found that changes in the expression of P-Ser^249^ and Thr^252^RB paralleled those of cyclin D1 (Figure 8B).

## DISCUSSION

This study revealed a significant decrease in the number of B2R/CD34 double positive cells in patients with DM. This decrease was inversely correlated with high plasma levels of MPO and BK, which were protective against reactive oxygen species (ROS)-induced cell senescence through B2R-dependent, AKT/Cyclin D1/RB and epidermal growth factor receptor (EGFR)/Cyclin D1/RB signaling pathways. This research is the first to demonstrate that oxidative stress impairs the expression of B2R on EPCs both *in vitro* and *ex vivo*, and conversely that BK suppresses ROS-induced senescence via a B2R-dependent pathway.

Oxidative stress plays a key role in various diseases, including DM and coronary artery disease (CAD) [12]. Elevated production of oxygen radicals is the defining characteristic of oxidative stress, which can injure tissues and organs and promote EPC senescence via activation of the ROS signaling cascade [13]. As a result, cellular senescence is thought to be a reflection of systemic dysfunction caused by oxygen radicals that lead to cell cycle arrest and a failure to repair cell injury.

B2R is a critical cell surface receptor molecule that is activated by BK and expressed on numerous cells, including EPCs that regulate cell proliferation and injury repair [14]. For example, low B2R expression is associated with inhibition of cell proliferation [15]. BK binds to both B1 (B1R) and B2 receptor. B1R is an inducible receptor that is elevated during inflammation and cell stress. Previous research has demonstrated that BK elicits its protective effects through recruitment of circulating CD34^+^ cells [16] and inhibition of H_2_O_2_-induced EPC senescence [17] through the B2R-mediated signaling pathway. In an aging rat model, the cardioprotective actions of bradykinin are physiologically mediated via B2R, whereas B1R is induced by tissue damage, which suggests that age-related decreases in B2R protein levels may leave the heart vulnerable to ischemic damage, and that increased B1R expression and activity may represent a compensatory reaction in aging hearts [18]. It can be concluded that BK inhibits H_2_O_2_-induced senescence of endothelial progenitor cells through the B2R signaling pathway. Furthermore, studies in animal models revealed that deficiencies in B2R function accelerate cell senescence and that B2R is strongly associated with P53 expression, as B2R knockout diabetic mice are resistant to oxidative stress-induced mitochondrial injury and show high expression of the tumor suppressor gene P53 [19]. A previous study showed that coronary artery disease is associated with down-regulation of B2R expression by circulating EPCs [20], which is supported by our research showing that B2R expression is lower on circulating CD34^+^ cells from patients with diabetes and is accompanied by higher degrees of oxidative stress compared with healthy subjects. These results suggest that oxidative stress caused by DM impairs B2R expression on circulating EPCs. Furthermore, we found that oxidative stress caused by oxygen radicals impairs the expression of B2R on EPCs and leads to EPC senescence. Therefore, we hypothesize that B2R is the key element that regulate oxidative stress-induced EPC senescence.

BK is the endogenous ligand of B2R and has protective effects that inhibit the senescence of endothelial cells by suppression of oxidative stress via activation of B2R [21, 22]. These findings were confirmed by our research, which demonstrated that BK inhibits the senescence of hEPCs treated with H_2_O_2_ by decreasing production of oxygen radicals via a B2R-dependent pathway.

A great number of studies have revealed that BK activates the PI3K/AKT pathway following binding to B2R and that it elicits its protective effect in part via the B2R/PI3K/AKT signal pathway [23]. Specifically, PI3K/AKT is activated by BK via B2R in cardiomyocyte cells, which then stimulates the AKT/eNOS pathway to protect hEPCs from oxidative stress and senescence [24, 25]. In this study, our results have confirmed that BK inhibition of oxidative stress and hEPC senescence is at least partly due to activation of the PI3K/AKT pathway via B2R. However, our study has also exposed a deeper mechanism involving the EGFR receptor tyrosine kinase. Several studies have shown that G protein-coupled receptors (GPCR) can transactivate EGFR and mediate several biological effects, including cell proliferation. Furthermore, BK mediation of cell proliferation in corneal fibroblasts depends on EGFR transactivation [26]. Activation of the EGFR signaling pathway can promote the cell cycle in late G1 [27]. In addition, EGFR signaling is reported to protect skeletal myoblasts from oxidative stress [28]. Consequently, the EGFR signaling pathway has protective effects that inhibit cell cycle arrest and oxidative stress. In our study, an EGFR antagonist blocked the protective effects of BK independent of AKT phosphorylation. This phenomenon suggests that BK may stimulate EGFR by transactivation via B2R to regulate in part hEPC senescence. However, this possibility will require additional experiments in the future to determine the specific factor that primarily regulates senescence.

The generation of senescence involves complicated signaling pathways and a variety of associated molecules that have been investigated in tumor cells, fibrosis cells, and immortalized cell lines. Currently, 2 major mechanisms, the P53/P21/cyclin/RB and P16/cyclin/RB signaling pathways, have been shown to contribute to cell senescence [29–31]. RB is a key molecule involved in both of these pathways that has the ability to stimulate cells to enter S phase or to remain in G0/G1 phase via phosphorylation and dephosphorylation activities, resulting in either RB release of or binding to the transcription factor E2F [32–34]. Additionally, DNA damage and senescence have been found to occur as a result of RB dephosphorylation in thyroid adenoma C cells [35], while deletion of RB can impair pancreatic cancer cell senescence, resulting in the acceleration of pancreatic cancer progression [36]. In progenitor cells, down-regulation of phosphorylated RB leads to doxorubicin-induced cardiac progenitor cell senescence [37]. Elevated expression of cyclin D1 leads to RB phosphorylation cell proliferation [38], while down-regulation of cyclin D1 and low RB phosphorylation lead to cell cycle arrest and senescence [39–41]. Oxidative stress is a promotional factor that leads to senescence. Cyclin D1 and RB are downstream proteins involved in the pathway of oxidative stress-induced senescence and oxidative stress can suppress cyclin D1 expression [42], but there are no reports that inhibition of cyclin D1 and RB phosphorylation mediates intracellular levels of oxygen radicals. However, the current understanding of the mechanism by which BK mediates anti-senescence pathways in CD34^+^ hEPCs is incomplete. In this study, we demonstrate that BK can increase cyclin D1 expression and RB phosphorylation and then inhibit hEPC senescence via 2 signaling pathways. Based on our data and previous studies, cyclin D1 and RB phosphorylation inhibition may promote the oxidative stress-induced senescence of CD34^+^ hEPCs.

Moreover, other studies have reported that the GPCR/PI3K/AKT pathway can directly regulate cell cycle-associated proteins [43]. Our research confirmed that BK promotes expression of cyclin D1 through the B2R/AKT pathway to inversely control RB via phosphorylation at Ser 249 and Thr 252. In addition, we found that BK stimulates EGFR activity via GPCR transactivation, and then further activates cyclin D1 to promote the RB phosphorylation. We suspect that oxidative stress inhibits RB phosphorylation, promoting senescence, while BK suppresses senescence via the hEPC PI3K/AKT signaling pathways. However, the signaling molecules involved in EGFR transactivation by GPCR remain uncertain. Previous studies have shown that various factors participate in the progression of transactivation, including Src [44], ADAM-17 [45], integrin α5β1 [46], and both collagenase-2 and -3 [47], which activate various signaling pathways involving different signaling molecules in different cells, making the identification of one responsible pathway quite challenging. Despite this study and previous research, the messenger molecule involved in EGFR transactivation by GPCR in hEPCs remains unknown. Additional research is required to identify the messenger participating in EGFR transactivation via GPCR.

## MATERIALS AND METHODS

### Determination of B2R and plasma myeloperoxidase levels in blood samples from patients with DM

Blood samples from 13 patients with DM treated at the ZhongDa hospital, along samples from 13 age- and gender-matched healthy controls, were collected in EDTA-coated tubes. The plasma was then separated, collected in EP tubes, and then stored at −40°C for later analysis. Mononuclear cells were isolated on hydroxypropyl methylcellulose (HaoYang, China) by centrifugation at 500 × g for 20 min. Afterwards, they were fixed in 1% paraformaldehyde and then permeabilized using 0.1% triton X-100 containing 0.5% BSA. The cells were then incubated with specific rabbit, anti-human B2R antibodies (Abcam, UK) on ice for 1 hour, followed by incubation with a PE-conjugated donkey anti-rabbit secondary antibody (Santa Cruz, USA) and a fluorescein isothiocyanate (FITC)-conjugated anti-human CD34 antibody (BD Biosciences, USA) for 1 hour. Stained cells were washed 3 times with PBS and then analyzed using a FACScan flow cytometer (Becton Dickinson, USA) to detect B2R labeling. The mononuclear cells were gated according to forward scatter and side scatter. The CD34^+^ cells were defined as the FITC-positive cell group. CD34^+^ cells were then gated and B2R^+^ cells were identified as PE-positive cells from among the CD34^+^ cells.

The concentration of plasma MPO, which reflects oxidative stress level, was determined by assay using a human MPO ELISA Kit (Boster, China) to determine the degree of *in vivo* oxidative stress in patients with diabetes and healthy controls.

### Human EPC culture and characterization

Human umbilical cord blood was obtained from ZhongDa Hospital in accordance with the Medical Ethics Committee of ZhongDa Hospital Affiliated with Southeast University (approval ID: 2013ZDSYLL108.1) and performed according to the Declaration of Helsinki. The cord blood was diluted 1:1 ratio in phosphate-buffered saline (PBS). The MNC fraction was then obtained via centrifugation (500g for 15 min) using a Lymphoprep density gradient (Sigma, USA), washed twice in PBS, and then centrifuged at 300g for 10 min. The cell pellet was suspended in endothelial basal growth medium (EBM-2) supplemented with EGM-2 MV SingleQuots (Lonza, USA) and 5% heat inactivated fetal bovine serum (FBS). The solution was plated in a T-25 culture flask coated with 10 μg/ml human plasma fibronectin (FN, Millipore, USA). After 96 hours, the unbound cells were removed and the bound cell fraction was maintained in culture using EGM-2, with spindle-shaped cells observed after 7 days. Colonies of endothelial-like cells grew until confluence and then were trypsinized and plated uniformly in new T-25 culture flasks as a first passage. The cell culture medium was changed every 3 days, and cells were passaged at a ratio of 1:2 when the cells reached 80% confluence. Subsequent passages were performed similarly, and the isolated hEPCs from passages 3 ∼ 6 were used in the study.

hEPCs were primarily characterized by phase contrast microscopy to evaluate cobblestone morphology. The cells were incubated with 1, 1′-dioctadecyl 3,3,3′,3-tetramethylindocarbocyanine-labeled, acetylated low-density lipoprotein (DiI-acLDL; Invitrogen, USA) for 4 hours at 37°C. Lectin binding was analyzed using fluorescein isothiocyanate (FITC)-conjugated UEA-1-lectin (Sigma, USA) to confirm the expression of endothelial cells marker lectin, and the cells were examined under a fluorescent microscope (Nikon, Japan). Immunofluorescence-based flow cytometry (Abcam, UK) was also utilized to determine the expression of the progenitor lineage markers CD34 and CD105 (both BD Biosciences, USA), the endothelial lineage marker KDR (BD Biosciences, USA), and the leukocyte marker CD45 (BD Biosciences).

### Determination of H_2_O_2_-induced senescence

hEPCs were treated with either H_2_O_2_ alone or BK plus H_2_O_2_ in 5% FBS-containing medium. Cells were incubated with BK at either 0.1 or 1.0 nM for 30 min immediately before H_2_O_2_. To label senescent cells, SA-Gal staining was performed 12 h after H_2_O_2_ treatment as previously described [48]. Briefly, cells were washed in PBS and fixed in 2% formaldehyde and 0.2% glutaraldehyde for 5 min at room temperature, then washed and incubated overnight at 37°C with SA-Gal staining solution (150 mM NaCl, 2 mM MgCl_2_, 5 mM K_4_[Fe(CN)_6_], and 5 mM K_3_[Fe(CN)_6_], in 40 mM citric acid/sodium phosphate dibasic at pH 6.0, containing 1 mg/mL of 5-bromo-4-chloro-3-indolyl-Dgalactoside). Light microscopy images were captured using an inverted microscope at 200× magnification and senescent cells were counted per microscopic field.

### Detection of intracellular oxygen radicals with a dichlorofluorescein diacetate probe

This experiment attempted to confirm that H_2_O_2_-induced senescence is caused by oxidative stress. Twenty-four hours after seeding cells in 24-well clusters, hEPCs were exposed to 300 μM H_2_O_2_ in 5% FBS-containing medium to induce production of intracellular oxygen radicals. BK (0.1 nM or 1.0 nM) was administered 30 min before the addition of H_2_O_2_. Twelve hours after initiating H_2_O_2_ exposure, cells in each well were washed 3 times in PBS, and then incubated in FBS-free medium (1 mL per well). A 10 μM DCFH-DA probe was added immediately and then incubated at 37°C for 20 min. Intracellular oxygen radicals were then determined using a fluorescence microscope at an absorption wave length of 488 nm and an emission wave length of 530 nm. There was no significant difference between 0.1 and 1.0 nM BK (*p* > 0.05), for this and the previous assay, so lower concentration was used for all remaining assays.

### Cell senescence PCR array

RT^2^ Profiler PCR Arrays (PAHS-050A, SABiosciences, Germany) were used to determine the senescence-associated molecular signal pathways. One well each of cells treated with either 300 μM H_2_O_2_ or 300 μM H_2_O_2_ plus 0.1 nM BK in 5% FBS-containing medium for 12 hours were collected and total RNA was extracted and cDNA synthesized as described below. The analyzed genes are described in Supplemental Table 2.

### Determination of the effect of a B2 receptor antagonist or PI3K and EGFR signaling pathway inhibitors on BK inhibition of oxidative stress-induced senescence

The B2R antagonist HOE-140 (150 nM), PI3K antagonist LY-294002 (10 μM), and EGFR antagonist AG1478 (10 μM) were added to block the B2R, PI3K, and EGFR separately for 5 min before the addition of BK. hEPCs were treated with H_2_O_2_ alone or 0.1 nM BK plus H_2_O_2_ as described above. SA-Gal staining was then performed and light microscopy images were taken on an inverted microscope (Nikon, Japan) at 200× magnification, and the number of senescent cells was counted per microscopic field. For detecting intracellular oxygen radicals, the DCFH-DA probe was used as described above and analyzed using a fluorescence microscope at an absorption wavelength of 488 nm and emission wavelength of 530 nm.

### Transfection of human B2 receptor siRNA and its effect on BK inhibition of oxidative stress-induced senescence

Cells were seeded in 6- or 24-well clusters or a T-25 culture flask and then incubated at 37°C in 5% CO_2_ until 80% confluent. B2R siRNA was purchased from Santa Cruz, USA. siRNA transfection solution was prepared according to directions provided by Santa Cruz to make a siRNA concentration of 400 nM. The cells were washed once with siRNA transfection medium (Santa Cruz, USA). Then, the appropriate siRNA transfection medium and siRNA transfection solution was added to each well. The cells were then incubated for 6 hours at 37°C in 5% CO_2_. The transfection mixture was then removed and replaced with normal growth medium and incubated for an additional 24 hours. The cells were then treated with either H_2_O_2_ alone or 0.1 nM BK plus H_2_O_2_ as described above. SA-Gal staining was then used to determine senescence and DCFH-DA probes visualized using a fluorescence microscope at an absorption wavelength of 488 nm and emission wavelength of 530 nm as described above.

### hEPC expression of B2R by flow cytometry

Cells were treated with H_2_O_2_ or H_2_O_2_ plus BK with or without signaling pathway inhibitors as described above, and then dissociated from the culture flask by trypsinization and transferred to an EP tube. After cell fixation in 1% paraformaldehyde and permeabilization in 0.1% triton X-100 containing 0.5% BSA, cells were incubated with specific rabbit anti-human B2R antibodies (Abcam, UK) on ice for 1 hour, washed 3 times with PBS, and then incubated with a PE-conjugated donkey anti-rabbit second antibody for 1 hour. B2R expression was quantified on a FACScan flow cytometer (Becton Dickinson, USA) with cells cultured in normal growth medium used as control.

### RNA extraction and RT-PCR

After cells with or without exposure to signaling pathway inhibitors were treated with H_2_O_2_ or BK plus H_2_O_2_ as described above, the cells were lysed using TRIzol Reagent (Invitrogen, USA). Total RNA was extracted according to the cell RNA extraction protocol offered by Invitrogen and cDNAs were synthesized using the PrimeScript^TM^ RT reagent kit with gDNA Eraser (TAKARA, Japan). RT-PCR was performed using B2R primers (sense: 5-TGCTGCTGCTATTCATCATC-3; antisense: 5-CCAGTCCTGCAGTTTGTGAA-3), 18S rRNA primers (sense: 5-CATGCTAACTAGTTACGCGACC-3; antisense: 5-GAGCAATAACAGGTCTGTGATG-3) and RB primers (sense: CGTGCGCTCTTGAGGTTGTAA; antisense: TTGGTCCTTCTCGGTCCTTTG). After RT (50°C, 30 min), hot start (94°C, 15 min), and 40 – 42 cycles of PCR (94°C, 1 min; 52.5°C, 1 min; 72°C, 1 min), RB and B2R mRNA expression was normalized to 18S rRNA and calculated as 2^−ΔΔCt^.

### Western blot analysis

For Western blot analysis, cells were briefly washed twice in cold PBS and incubated for 10 min on ice in a lysis buffer (50 mM Tris (pH 8.0), 150 mM NaCl, 0.02% sodium azide, 0.2% SDS, 100 μg/mL phenylmethylsulfonylfluoride [PMSF, Sigma, USA], 50 μL/ml aprotinin, 1% octylphenoxypolyethoxyethanol 630, 100 mM NaF, 0.5% sodium deoxycholate, 0.5 mM EDTA, 0.1 mM ethylene glycol tetraacetic acid). The lysates were centrifuged at 12,000g for 5 min, and then the supernatants were collected to obtain the cytosolic proteins and stored at − 80°C. To obtain the nuclear proteins, the lysis buffer (26% glycerol [pH 7.9], 5 mM 2-[4-(2-Hydroxyethyl)-1-piperazinyl]ethanesulfonic acid (HEPES), 1.5 mM MgCl_2_, 0.2 mM ethylenediaminetetraacetic acid, 0.5 mM DL-dithiothreitol, 300 mM NaCl) was added to the precipitation from the previous step. Samples were vortexed for 30s out of every 2 min for 30 min, then centrifuged at 12000g for 10 min. The supernatant was collected and stored at −80°C. Protein concentrations were measured using the BCA assay kit (Pierce, USA). Western blots were performed using cytosolic protein samples to detect AKT, phosphorylated Ser^473^-AKT (P-Ser^473^AKT) using rabbit monoclonal anti-human AKT antibodies or a rabbit monoclonal anti-human phosphorylated Ser^473^-AKT antibody (Cell Signaling Technology, USA). Nuclear proteins were used to detect RB, phosphorylated Ser^249^, Thr^252^ RB (P-Ser^249^ and Thr^252^ RB), and cyclin D1 using rabbit monoclonal anti-human phosphorylated Ser^249^, Thr ^252^RB antibodies and a rabbit monoclonal anti-human cyclin D1 antibody (Cell Signaling Technology, USA). GAPDH was used as a loading control (KangCheng Bio-tech, China). After primary antibody incubation, blots were incubated with the appropriate secondary horseradish peroxidase conjugate mouse monoclonal anti-rabbit antibody (Boster, China). Each membrane was washed and then developed using the SuperSignal chemiluminescent substrate (Pierce, USA).

### Statistical analyses

For all experiments, data was analyzed using either a Student's *t*-test or Bonferroni's test, with values expressed as the mean ± SEM. All statistical analyses were performed using SPSS software (SAS Institute Inc, USA), with a *p* value < 0.05 considered to be statistically significant.

## SUPPLEMENTARY TABLES


