# Supplementary material for: Bradykinin inhibits oxidative stress-induced senescence of endothelial progenitor cells through the B2R/AKT/RB and B2R/EGFR/RB signal pathways
Source: Oncotarget. 2015 Aug 24;6(28):24675–89. doi: 10.18632/oncotarget.5071 (PMC4694787; doi:10.18632/oncotarget.5071)
Supplement: Supplementary file 1 [file oncotarget-06-24675-s001.pdf]

SUPPLEMENTARY TABLES

Supplementary Table S1: Gene involved in PCR array that changed more than 1.2 fold

Gene name	Fold change
RPLP0	-1.22
IGFBP7	-1.25
CDKN1A	-1.33
GAPDH	1.27
CALR	-1.52
SERPINE1	-1.55
CDK4	-1.24
EGR1	-1.32
CCND1	-1.32
AKT1	-1.22
CCNB1	1.31
TERF2	-1.2
E2F3	-1.27
MAP2K3	-1.25
ETS1	-1.24
NFKB1	-1.33
COL1A1	1.79
COL3A1	1.32
CCNA2<br>CITED2	1.27<br>1.22
CDKN2A	-1.20
CDKN1B	-1.23
TBX2	-1.27
CDC25C	-1.34
CDKN2B	1.47
IGFBP3	1.22
MAP2K6	-1.21
GADD45A	-2.68
IGFBP5	-2.89
ING1	2.94
RB1	-176.15
RBL1	35.58
TWIST1	-2.81

Supplementary Table S2: Analyzed gene involved in PCR array

Gene name	Position	Gene bank	Description
ABL1	A01	NM_005157	C-abl oncogene 1, non-receptor tyrosine kinase
AKT1	A02	NM_005163	V-akt murine thymoma viral oncogene homolog 1
ALDH1A3	A03	NM_000693	Aldehyde dehydrogenase 1 family, member A3
ATM	A04	NM_000051	Ataxia telangiectasia mutated
BMI1	A05	NM_005180	BMI1 polycomb ring finger oncogene
CALR	A06	NM_004343	Calreticulin
CCNA2	A07	NM_001237	Cyclin A2
CCNB1	A08	NM_031966	Cyclin B1
CCND1	A09	NM_053056	Cyclin D1
CCNE1	A10	NM_001238	Cyclin E1
CD44	A11	NM_000610	CD44 molecule (Indian blood group)
CDC25C	A12	NM_001790	Cell division cycle 25 homolog C (S. pombe)
CDK2	B01	NM_001798	Cyclin-dependent kinase 2
CDK4	B02	NM_000075	Cyclin-dependent kinase 4
CDK6	B03	NM_001259	Cyclin-dependent kinase 6
CDKN1A	B04	NM_000389	Cyclin-dependent kinase inhibitor 1A (p21, Cip1)
CDKN1B	B05	NM_004064	Cyclin-dependent kinase inhibitor 1B (p27, Kip1)
CDKN1C	B06	NM_000076	Cyclin-dependent kinase inhibitor 1C (p57, Kip2)
CDKN2A	B07	NM_000077	Cyclin-dependent kinase inhibitor 2A (melanoma, p16, inhibits CDK4)
CDKN2B	B08	NM_004936	Cyclin-dependent kinase inhibitor 2B (p15, inhibits CDK4)
CDKN2C	B09	NM_078626	Cyclin-dependent kinase inhibitor 2C (p18, inhibits CDK4)
CDKN2D	B10	NM_001800	Cyclin-dependent kinase inhibitor 2D (p19, inhibits CDK4)
CHEK1	B11	NM_001274	CHK1 checkpoint homolog (S. pombe)
CHEK2	B12	NM_007194	CHK2 checkpoint homolog (S. pombe)
CITED2	C01	NM_006079	Cbp/p300-interacting transactivator, with Glu/Asp-rich carboxy-terminal domain, 2
COL1A1	C02	NM_000088	Collagen, type I, alpha 1
COL3A1	C03	NM_000090	Collagen, type III, alpha 1
CREG1	C04	NM_003851	Cellular repressor of E1A-stimulated genes 1
E2F1	C05	NM_005225	E2F transcription factor 1
E2F3	C06	NM_001949	E2F transcription factor 3
EGR1	C07	NM_001964	Early growth response 1
ETS1	C08	NM_005238	V-ets erythroblastosis virus E26 oncogene homolog 1 (avian)
ETS2	C09	NM_005239	V-Ets erythroblastosis virus E26 oncogene homolog 2 (avian)
FN1	C10	NM_002026	Fibronectin 1
GADD45A	C11	NM_001924	Growth arrest and DNA-damage-inducible, alpha
GLB1	C12	NM_000404	Galactosidase, beta 1

(*Continued*)

Gene name	Position	Gene bank	Description
GSK3B	D01	NM_002093	Glycogen synthase kinase 3 beta
HRAS	D02	NM_005343	V-Ha-ras Harvey rat sarcoma viral oncogene homolog
ID1	D03	NM_002165	Inhibitor of DNA binding 1, dominant negative helix-loop-helix protein
IFNG	D04	NM_000619	Interferon, gamma
IGF1	D05	NM_000618	Insulin-like growth factor 1 (somatomedin C)
IGF1R	D06	NM_000875	Insulin-like growth factor 1 receptor
IGFBP3	D07	NM_000598	Insulin-like growth factor binding protein 3
IGFBP5	D08	NM_000599	Insulin-like growth factor binding protein 5
IGFBP7	D09	NM_001553	Insulin-like growth factor binding protein 7
ING1	D10	NM_005537	Inhibitor of growth family, member 1
IRF3	D11	NM_001571	Interferon regulatory factor 3
IRF5	D12	NM_001098629	Interferon regulatory factor 5
IRF7	E01	NM_001572	Interferon regulatory factor 7
MAP2K1	E02	NM_002755	Mitogen-activated protein kinase kinase 1
MAP2K3	E03	NM_002756	Mitogen-activated protein kinase kinase 3
MAP2K6	E04	NM_002758	Mitogen-activated protein kinase kinase 6
MAPK14	E05	NM_001315	Mitogen-activated protein kinase 14
MDM2	E06	NM_002392	Mdm2 p53 binding protein homolog (mouse)
MORC3	E07	NM_015358	MORC family CW-type zinc finger 3
MYC	E08	NM_002467	V-myc myelocytomatosis viral oncogene homolog (avian)
NBN	E09	NM_002485	Nibrin
NFKB1	E10	NM_003998	Nuclear factor of kappa light polypeptide gene enhancer in B-cells 1
NOX4	E11	NM_016931	NADPH oxidase 4
PCNA	E12	NM_182649	Proliferating cell nuclear antigen
PIK3CA	F01	NM_006218	Phosphoinositide-3-kinase, catalytic, alpha polypeptide
PLAU	F02	NM_002658	Plasminogen activator, urokinase
PRKCD	F03	NM_006254	Protein kinase C, delta
PTEN	F04	NM_000314	Phosphatase and tensin homolog
RB1	F05	NM_000321	Retinoblastoma 1
RBL1	F06	NM_002895	Retinoblastoma-like 1 (p107)
RBL2	F07	NM_005611	Retinoblastoma-like 2 (p130)
SERPINB2	F08	NM_002575	Serpin peptidase inhibitor, clade B (ovalbumin), member 2
SERPINE1	F09	NM_000602	Serpin peptidase inhibitor, clade E (nexin, plasminogen activator inhibitor type 1), member 1
SIRT1	F10	NM_012238	Sirtuin 1
SOD1	F11	NM_000454	Superoxide dismutase 1, soluble
SOD2	F12	NM_000636	Superoxide dismutase 2, mitochondrial

(*Continued*)

Gene name	Position	Gene bank	Description
SPARC	G01	NM_003118	Secreted protein, acidic, cysteine-rich (osteonectin)
TBX2	G02	NM_005994	T-box 2
TBX3	G03	NM_016569	T-box 3
TERF2	G04	NM_005652	Telomeric repeat binding factor 2
TERT	G05	NM_198253	Telomerase reverse transcriptase
TGFB1	G06	NM_000660	Transforming growth factor, beta 1
TGFB1I1	G07	NM_015927	Transforming growth factor beta 1 induced transcript 1
THBS1	G08	NM_003246	Thrombospondin 1
TP53	G09	NM_000546	Tumor protein p53
TP53BP1	G10	NM_005657	Tumor protein p53 binding protein 1
TWIST1	G11	NM_000474	Twist homolog 1 (Drosophila)
VIM	G12	NM_003380	Vimentin
ACTB	H01	NM_001101	Actin, beta
B2M	H02	NM_004048	Beta-2-microglobulin
GAPDH	H03	NM_002046	Glyceraldehyde-3-phosphate dehydrogenase
HPRT1	H04	NM_000194	Hypoxanthine phosphoribosyltransferase 1
RPLP0	H05	NM_001002	Ribosomal protein, large, P0
HGDC	H06	SA_00105	Human Genomic DNA Contamination
RTC	H07	SA_00104	Reverse Transcription Control
RTC	H08	SA_00104	Reverse Transcription Control
RTC	H09	SA_00104	Reverse Transcription Control
PPC	H10	SA_00103	Positive PCR Control
PPC	H11	SA_00103	Positive PCR Control
PPC	H12	SA_00103	Positive PCR Control
